# Identification of Potential Drug Targets Implicated in Parkinson's Disease from Human Genome: Insights of Using Fused Domains in Hypothetical Proteins as Probes

**DOI:** 10.5402/2011/265253

**Published:** 2011-09-04

**Authors:** N. Rathankar, K. A. Nirmala, Varun Khanduja, H. G. Nagendra

**Affiliations:** ^1^Department of Bioinformatics, School of Bioengineering, SRM University, Kattankulathur, Tamil Nadu 603 203, India; ^2^Physics Department, Bangalore University, Jnanabharathi Campus, Bangalore 560 056, India; ^3^Department of Biotechnology, Sir M. Visvesvaraya Institute of Technology, Near Hunasamaranahalli, Via Yelahanka, Bangalore 562 157, India

## Abstract

High-throughput genome sequencing has led to data explosion in sequence databanks, with an imbalance of sequence-structure-function relationships, resulting in a substantial fraction of proteins known as hypothetical proteins. Functions of such proteins can be assigned based on the analysis and characterization of the domains that they are made up of. Domains are basic evolutionary units of proteins and most proteins contain multiple domains. A subset of multidomain proteins is fused domains (overlapping domains), wherein sequence overlaps between two or more domains occur. These fused domains are a result of gene fusion events and their implication in diseases is well established. Hence, an attempt has been made in this paper to identify the fused domain containing hypothetical proteins from human genome homologous to parkinsonian targets present in KEGG database. The results of this research identified 18 hypothetical proteins, with domains fused with ubiquitin domains and having homology with targets present in parkinsonian pathway.

## 1. Introduction

Hypothetical proteins basically are defined as “a protein coded by a gene with no known function based on its DNA sequence” [2]. Certain regions in hypothetical proteins are highly conserved between species in both composition and sequence. Proteins with such regions are annotated as conserved hypothetical proteins and range from 13% in *E. coli* and 14% in *Rickettsia prowazekii* to 40% in *Pyrococcus abyssi* and 47% in *Plasmodium falciparum* [3]. The human genome too has about 20% of them classified as hypothetical [4–6].

The function of such proteins can be predicted based on the arrangement of distinct domains [7] in them since this arrangement in proteomes reflects the fundamental evolutionary differences in their genomes [8]. But with proteins containing more than one domain, the general function can only be suggested. The difficulty one observes in predicting a protein's function based on domains alone would be when there are no clear cut boundaries between any two domains. Proteins with appreciable overlap in their domain boundaries are known as fused domain containing proteins or chimeric proteins. Such proteins are formed by the process of gene duplication and combination during evolution. Proteins containing such domains are created by joining two or more genes, which originally code for separate proteins [9]. Translation of this fusion gene results in a single polypeptide with functional properties derived from each of the original proteins [10]. Analysis of these fused domains in related genomes reveals the fact that fused domain proteins in eukaryotic genomes correspond to single, full-length proteins in prokaryotic genomes [11]. 

Proteins with fused domains [12] in a genome are likely to be involved in metabolic and signaling pathways [13]. A study by Chia and Kolatkar [14] illustrates that domain fusions can be used to predict protein-protein interactions. This method has proven to be effective in predicting functional links between proteins. Analysis of the structures of multidomain single-chain peptides in their study revealed that domain pairs located less than 30 residues apart on a chain share a physical interface, and their interactions are conserved. Apart from its normal functions, these multidomain-containing proteins are also implicated in several diseases. The bcr-abl fusion protein is a well-known example of an oncogenic fusion protein and is considered to be the primary oncogenic driver of chronic myelogenous leukemia [15]. A study on 70 positionally cloned human genes mutated in diseases found that a significantly high proportion of these “disease genes” contained several signaling domains including the DEATH domain and play active roles in cell signaling [16, 17]. 

Structural Classification of Proteins (SCOP) [18] suggests that these multidomain proteins can be classified based on the fold of a protein that contain two or more domains belonging to different classes. Based on this, SCOP 1.73 classifies the PDB structures with multidomains into 53 folds, which covers 1277 structures in total. A recent classification of multidomains on this SCOP database by Wang and Caetano-Anollés [19] broadly classifies them into five categories, namely, (i) single-domain proteins, (ii) single domain in multidomain proteins, (iii) domain repeats, (iv) domain repeats in multidomains, and (v) domain pairs. Interestingly it is observed that none of these classifications addressed the proteins containing fused/overlapping domain containing proteins. Hence, an attempt has been made by us in this paper to classify the multidomain proteins from the Human Hypothetical protein dataset into three major classes, namely,

- nonrepeating and unique domains,
- repeat and nonoverlapping domains, and
- overlapping/fused domains.

Further, as a case study, an in-depth analysis has been carried out to elucidate the roles of multidomain proteins involved in Parkinson's disease.

## 2. Materials and Methods

Characterizing the protein function in a proteome is a multistep process involving selection of homologs, building multiple sequence alignment, extracting relevant domain information, and then targeting them to the proteome using machine's learning algorithms such as Hidden Markov Models (HMMs), Support Vector Machines (SVMs), consensus sequences, and so forth, in order to denote their functional annotation. Hence, multiple sequence alignments from the CDD [20] database were used as targets to build HMMs. This approach has seen success in classifying human proteins with novel functions [21]. The protocol followed is briefed below.

### 2.1. Step 1: Extracting the Dataset of Multidomain Proteins

In order to extract the hypothetical proteins with multidomains, domain information from the CDD was used as a resource, and HMMs were built for all the 2009 domains present in the CDD using the HMMBUILD module of HMMER. These HMMs were used as targets to search against the hypothetical proteins database using the HMMSEARCH [22] module. Sequences with *e*-value less than 0.001 were only considered as meaningful targets, which resulted in a total of 1,777 sequences.

### 2.2. Step 2: Extracting Fused Domains Sequences from Multidomain Sequences

Of these 1777 protein sequences, 984 were with single domain, and 793 belonged to multidomain sequences. A parameter known as overlapping ratio (*L*), defined by

(1) $$L=\frac{\text{Overlapping}\;\text{length}}{\text{length}\;\text{of}\;\text{the}\;\text{larger}\;\text{domain}}$$

was calculated for all the 793 multidomain sequences. Thus, sequences with *L* = 0 denotes nonoverlapping multidomain proteins and that with *L* > 0 denotes multidomain proteins with fused domains. A cut-off value of *L* = 0.50 was chosen to extract more probable fused domain sequences from the multidomain sequence dataset. 

Thus, these calculations resulted into a total of 360 sequences with nonoverlapping domains (*L* = 0) and **433 **sequences with overlapping or fused domains (*L* > 0.5). Interestingly 20% of the domain fusions is prominent due to the three domains cd00053, cd00054, and cd00079.

### 2.3. Step 3: Clustering Domains Based on Overlap Data

**Figure 1:**
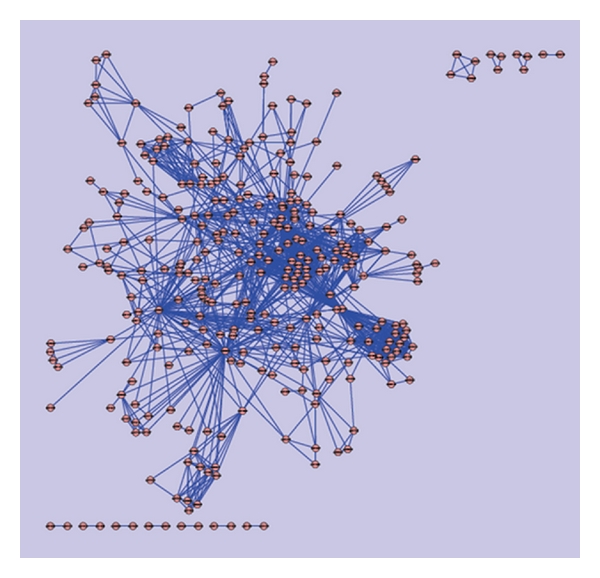
17 clusters in fused hypothetical proteins containing 36 domains.

Frequencies of the fused domains in the hypothetical proteins dataset was used as an input for clustering using a clustering software known as Cytoscape [23]. This yielded a total of 17 clusters (Figure 1), of which the largest cluster had a total of 36 domains resulting from 106 hypothetical sequences.

This cluster containing ubiquitin, ubiquitin-like & kinase motor domain(s) sequences were associated with diseases such as Alzheimer's, Von Hippel Lindau, juvenile parkinsonism, and spinocerebellar ataxia. In a similar way, domains in each cluster were analyzed by using their functional information from the CDD, and a table of these clusters with their functions are as shown in Table 1.

Table 1 indicates clearly that ubiquitin-like domains are involved in neurological disorders. Hence, clusters with fused ubiquitin domains were considered for further analysis, as they could become potential drug targets for a variety of neurodegenerative disorders [24]. Based on this criteria, sequences from the clusters 1, 2, 5, and 8 were selected. To investigate the role of multidomains in neurodegenerative diseases, fused ubiquitin domains related to Parkinson's disease were considered.

Parkinson's disease (PD) is a progressive disorder of the central nervous system affecting approximately one million people in the United States alone, wherein 50,000 new cases are reported annually [1]. Clinically, the disease is characterized by a decrease in spontaneous movements, gait difficulty, postural instability, rigidity, and tremor [25].

At the molecular level, the details regarding the genes that have been suggested to cause hereditary parkinsonism, and chromosomal loci associated with Parkinsonism in other families are as tabulated in Table 2. From Table 2, it is clear that ubiquitin/ubiquitin-like domains play a dominant role in the onset of Parkinson's disease. Hence, fused Ubiquitin domain sequences from clusters 1, 2, 5, and 8 were considered for a detailed investigation to ascertain their roles as well.

### 2.4. Step 4: Arrival of a Target Dataset for Parkinson's Disease

**Figure 2:**
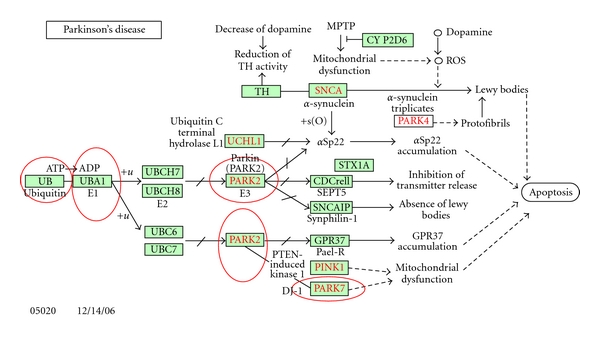
Parkinson's disease pathway from the KEGG disease database. Proteins encircled with red color are the ones having fused domains with ubiquitin domains (source: the KEGG disease pathway database).

In order to characterize these hypothetical proteins with fused domains, 18 sequences belonging to Parkinson's pathway were extracted from the KEGG database [26] (Figure 2) and were queried against the CDD [20]. Four (UB, UBA1, PARK2, and PARK7) out of eighteen sequences were observed to have fused ubiquitin domains. These sequences are highlighted in Figure 2, which is illustrated in the KEGG Parkinson's disease pathway.

### 2.5. Step 5: Extracting Relevant Homologues for Parkinson Diseased Targets

These four Parkinson's diseased sequences (UB, UBA1, PARK2 and PINK1) were searched against the sequences in four clusters (i.e., clusters numbered 1, 2, 5, and 8). 

A cut-off *e*-value of 1*e* − 04 was used as a filter to arrive at relevant hypothetical protein homologues. This search resulted into a total of 18 hypothetical sequences, which could be potential drug targets. A table representing the homologues with the sequences from the KEGG database is as shown in Table 3.

### 2.6. Step 6: Sequence Analysis of Hypothetical Proteins

**Cluster-1: (Ubiquitin, Ubiquitin-Like & Kinase Motor Domain(s)).**

**Figure 3:**
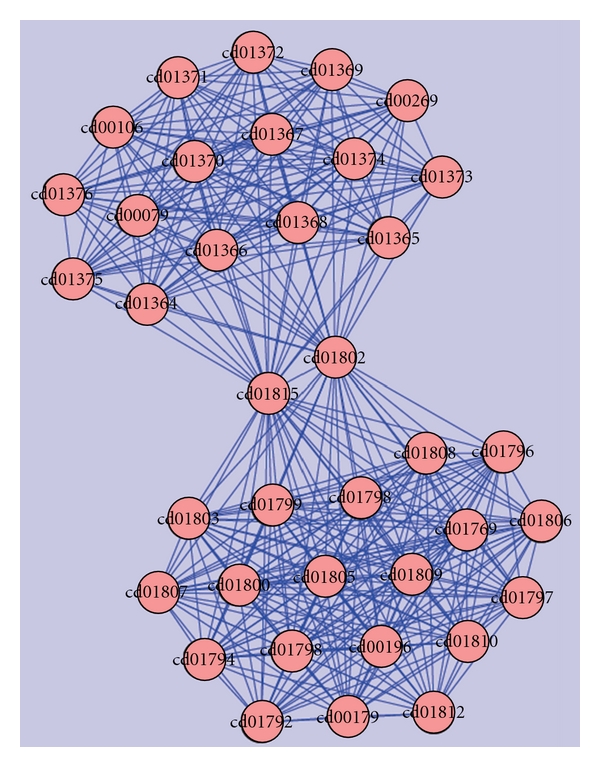
Domains in cluster-1.

The first is the largest cluster amongst the 17 clusters. This cluster (Figure 3) contained a total of 36 domains resulting from 106 hypothetical sequences. Sequences in this cluster were associated with neurodegenerative disorders such as Alzheimer's, juvenile parkinsonism, and spinocerebellar ataxia. A database search for the four Parkinson's diseased targets (UB, PARK2, UBA1, and PINK1) against the sequences in this cluster resulted in six hypothetical sequences from human genome, of which five were related to UB, three related to PARK2, and two homologous to both UB and PARK2 proteins.

**Figure 4:**
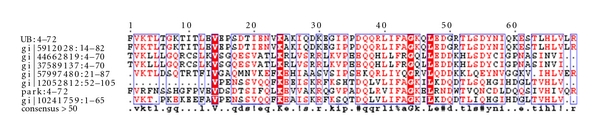
Multiple sequence alignment of the fused domains in 6 unique hypothetical proteins and their target sequences (PARK2 and ubiquitin sequence).

As illustrated in Table 4, the fusions between the domains cd00196, cd01796, and cd01089 are conserved in all these six hypothetical sequences and their respective protein targets. A pairwise comparison of these sequences with their targets are shown in Figure 4 to reiterate the same at the sequence level.

**Mutational Analysis**

**Figure 5:**
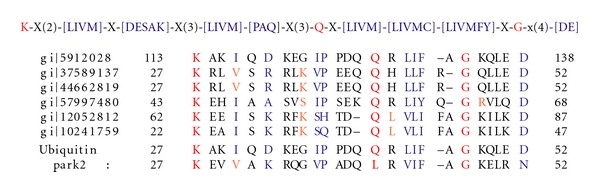
PROSITE signature PS00299 comparison in hypothetical sequences for a ubiquitin domain. Square brackets in the signature indicate the presence of either of the residues at that position, whereas the x(3) indicates any three amino acids. The red-colored residue indicates the strictly conserved residues, blue-colored ones indicates the residues present in the regular expression patterns, and the orange-colored ones indicate the mutant residues as observed from the mutant database.

Mutational analysis of these proteins was carried out using the PROSITE [27] signature PS00299 (Figure 5).

This signature of 26 residues, from the 27th position and to the 52nd position, is the characteristic of ubiquitin domain. Of the four Parkinson's disease homologs, PARK2 and the Ubiquitin protein (UB) have this signature. A comparison of this motif with the homologs of the parkinsonian targets was carried out, and the mutants were compared with the protein mutant database (PMD) [28] to infer the effects of such mutations. A table depicting the mutants in the ubiquitin domain with their altered functions is as shown (Table 5).

**Cluster-2**

**Figure 6:**
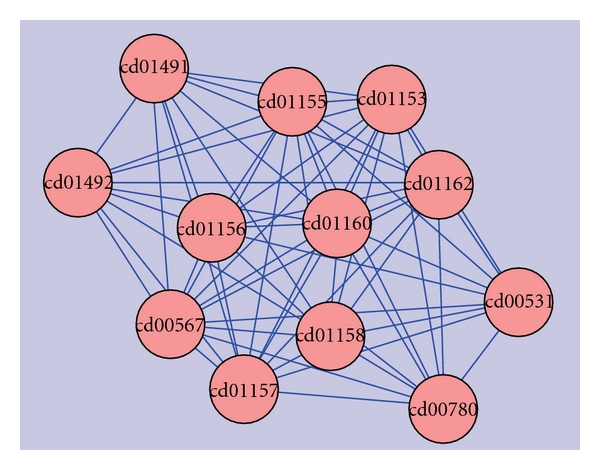
Domains in cluster-2.

**Figure 7:**
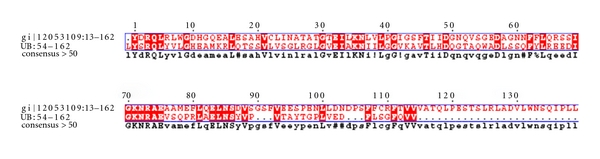
Pairwise sequence alignment of the fused domains between the ubiquitin sequence and its homolog hypothetical sequence (gi:12053109).

Cluster-2 (Figure 6) had 11 domains spanning a total 11 hypothetical protein sequences. Majority of the domains in cluster-2 were ubiquitin, NTF2, and ubiquitin-like domains. Sequences in this cluster were associated with fatty acid disorders. A database search for the four parkinson's diseased targets (UB, PARK2, UBA1, and PINK1) against the sequences in this cluster resulted in only one hypothetical protein from human genome. This hypothetical protein with domains cd01491 and cd01492 being fused, is seen to be conserved as observed in UBA1 (Table 6). A pairwise comparison of the hypothetical sequence (gi:12053109) with its parkinsonian homolog (UBA1) is as shown in Figure 7.

**Cluster-5**

**Figure 8:**
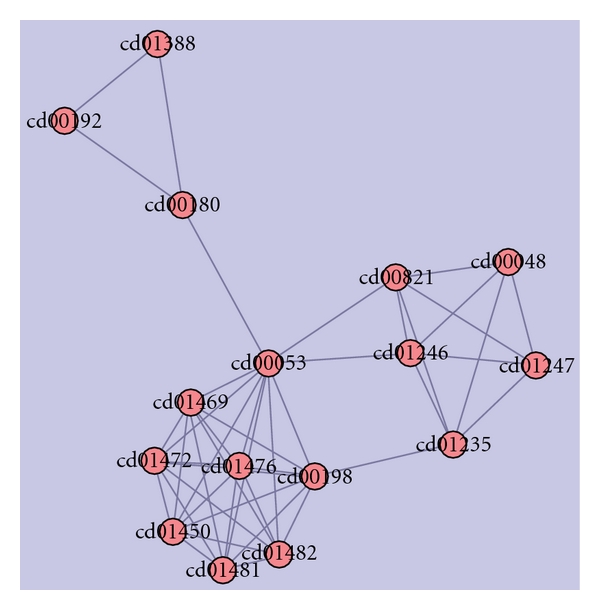
Domains in cluster-5.

**Figure 9:**
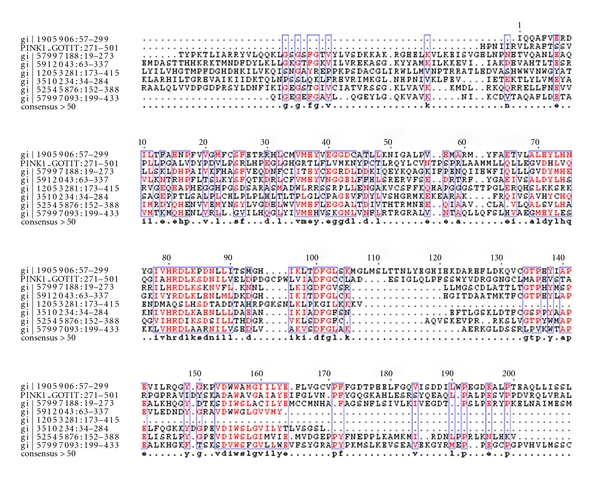
Multiple-sequence alignment of the fused domains in 7 unique hypothetical proteins and PINK1 sequence.

Cluster-5 had 16 domains spanning over 196 hypothetical protein sequences as depticted in Figure 8. Majority of the domains were PH, vWFA, and Ubiquitin-like domains. Sequences in this cluster were associated with von Willebrand disease, thrombotic thrombocytopenic purpura (TTP), hemolytic uremic syndrome (HUS), and ADAMTS13. A database search for the four parkinson's diseased targets (UB, PARK2, UBA1, and PINK1) against the sequences in this cluster resulted in seven hypothetical sequences from human genome, homologous to the parkinsonian target PINK1. The fusions between the domains cd00192 and cd00180 are conserved in all the hypothetical sequences and PINK1. Comparison of domain fusions in hypothetical sequences with their targets is represented in Table 7. A multiple sequence alignment of these sequences with PINK1 are shown in Figure 9.

**Cluster-8**

**Figure 10:**
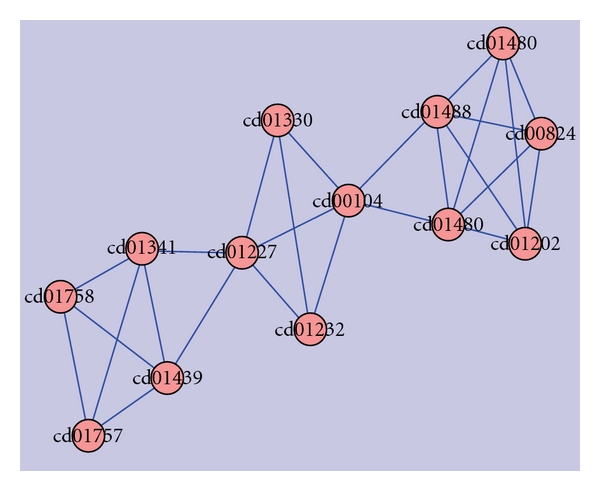
Domains in cluster-8.

Cluster-8 has 13 domains spanning 32 hypothetical protein sequences as shown in Figure 10. The functions of the majority of the domains were related to ubiquitin, PLAT, and ubiquitin-like domains. Sequences in this cluster were associated with neurological disorders.

**Figure 11:**
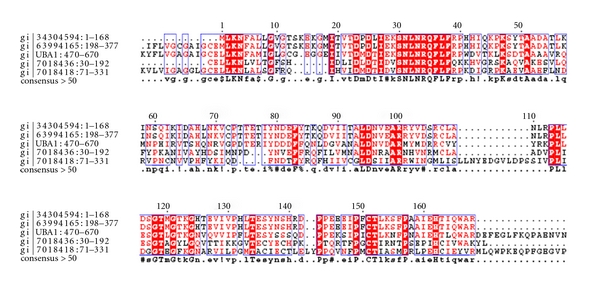
Multiple-sequence alignment of the fused domains in 4 unique hypothetical proteins and their target sequence (UBA1).

A database search for the four Parkinson's diseased targets (UB, PARK2, UBA1, and PINK1) against the sequences in this cluster resulted into four hypothetical sequences from human genome, homologous to the parkinsonian target PINK1. The fusions between the domains cd01488, cd01489 and cd01490 were observed to be conserved in all the hypothetical sequences and UBA1. Comparison of domain fusions in hypothetical sequences with their targets is represented in Table 8. A pairwise comparison of these sequences with their targets are shown in Figure 11.

## 3. Results and Discussions

This study was initiated to understand the diversity of functions in proteins with multiple-fused domains and to characterize the hypothetical proteins containing multiple-fused domains from human genome.

The approach involved characterizing hypothetical protein sequences (15480) based on identification of domains using the CDD database. This provided 1777 sequences with domains, of which 984 were single domains and 793 with multidomain sequences. Of these 793 sequences, 433 were multidomain-fused proteins. Frequencies of the 433 fused domain proteins were fed as an input for clustering using Cytoscape, which yielded a total of 17 clusters, as depicted in Figure 1. Four clusters amongst these 17 had ubiquitin fused-domain-containing sequences, which play an important role in a variety of neuropathological conditions including Parkinson's disease, Pick's disease, and Alzheimer's disease as indicated in Table 1. Ubiquitin domain consists of 76 amino acids and has been found in all eukaryotic cells. Apart from its use in protein degradation, ubiquitins are also involved in Parkinson's disease.

Parkinson's disease-related genes such as PARK2 and PINK1 has ubiquitin domains associated with them. Mutations in these sequences have prominently been associated with the onset of Parkinson's disease. As a case study, sequences in Parkinson's disease were used as basis to characterize the hypothetical proteins from the above-mentioned four clusters. From Table 2, it is clear that ubiquitin/ubiquitin-like domains play a dominant role in the onset of Parkinson's disease. Hence, fused ubiquitin domain sequences from clusters 1, 2, 5, and 8 were consid-ered for a detailed investigation to ascertain their roles as well. Similarity searches revealed 18 hypothetical proteins, homologous with the sequences implicated in Parkinson's disease, as shown in Table 3. Sequences in each of these clusters were then multiply aligned with the parkinsonian targets UB, UBA1, PARK2 & PINK1, to ascertain the presence of key patterns/signatures amongst them. As illustrated in Figures 4, 7, 9, and 11, conservation of residues amongst hypothetical proteins and Parkinson's sequences is highlighted.

## 4. Conclusions

We herewith conclude that the presence of fused domain as a signal in ubiquitin-containing proteins from parkinsonian targets is used as a probe to identify and characterize the functions of 18 hypothetical sequences, which could be used as lead drug targets for designing drugs in Parkinson's disease from human genome.

## Figures and Tables

**Table 1:** Summary of 17 clusters along with their involvement in diseases and major functions. Words in bold indicate neurodegenerative disorders and the role of ubiquitin domains in these disorders.

Cluster no.	No. of domains	Function of domains	No. of sequences	Disease implication
1	36	**Ubiquitin** & kinase motor domain	106	**Alzheimer's**, Von Hippel Lindau, **juvenile parkinsonism, spinocerebellar ataxia**
2	11	NTF2, **Ubiquitin,** CoA dehydrogenase	11	Fatty acid disorders
3	20	Myosin Motor	135	Familial hypertrophic cardiomyopathy, **neuromuscular disorders**
4	9	Cyclophilin	3	Immunosuppression, antiviral activity
5	16	vWFA & PH, **Ubiquitin**	196	Von Willebrand disease, thrombotic thrombocytopenic purpura (TTP)
6	6	PH & PTB	70	Cardiovascular diseases
7	10	tRNA synthase	15	Aminoacyl tRNA synthetase- charcot-Marie-Tooth disease type 2D, Mobius syndrome, cardiac disorders
8	13	**Ubiquitin **& PLAT	32	**Neurological disorders**
9	5	RNA binding	1	Myxoid liposarcoma, sars
10	5	SIR 2	3	**Neurological disorders**
11	5	HMG box	10	**Alzheimer's disease**, heart diseases, diabetes and cancer
12	4	PI3K	2	Cancer, diabetes and respiratory
13	4	Sm & Sm-like	8	Inflammatory bowel disease, Salla disease, diabetes
14	3	Methyl-CpG and PH	3	Cancer**, Down syndrome-like Alzheimer's**, Wiskott-Aldrich syndrome, B-cell chronic lymphocytic leukemia
15	3	EVH1	1	Wiskott-Aldrich syndrome
16	3	CGH & Ntn	1	Lysosomal storage disease and **progressive neurodegeneration,** Farber's disease, and **Alzheimer's Disease**
17	3	Nidogen, thyroglobulin type 1	6	Human gastrointestinal cancer, cancer, acute leukemia, heart diseases

**Table 2:** A description of genes, domains, and type of inheritance for Parkinson's disease (source: Nirit Lev and Melamed [1]).

Gene/locus/assignment	Domains present	Inheritance	Age of onset
a-Synuclein/SNCA/PARK1 & 4	Synuclein/**ubiquitin**-like	Autosomal dominant/susceptibility	Early/late
Parkin/PRKN/PARK2	Parkin and **ubiquitin**	Autosomal recessive/possible susceptibility	Juvenile/early
Ubiquitin C-terminal hydrolase/UCH-L1/PARK5	Peptidase, **ubiquitin **	Autosomal dominant/susceptibility	Late
DJ-1/DJ-1/PARK7	GATase/**ubiquitin**-like	Autosomal recessive	Early

**Table 3:** Hypothetical proteins homologous to KEGG sequences with fused domains in Parkinson's disease pathway.

Sl. no.	The KEGG protein ID	Gi ID	Cluster no.	No. of Hypothetical proteins	Gi ID
1	UB	11024714	1	1	5912028
2	PARK2	4758884	1	5	10241759
					12052812
					44662819
					57997480
					37589137
3	UBA1	23510338	2	1	12053109
			8	4	7018418
					7018436
					34304594
					63994165
4	PINK1	14165272	5	7	1905906
					3510234
					5912043
					12053281
					52545876
					57997093
					57997188

**Table 4:** Conservation of domain fusions in Parkinson's disease targets and human hypothetical sequences.

KEGG sequence	Hypothetical protein's Gi IDs	Region of domain fusion with the target					Sequence identity in the fused region (%)	
		Cd00196	Cd01769	Cd01809	UB	PARK2	UB	PARK2
UB	**5912028**	14–82,	14–82,	11–82,	4–72,		98	
		90–158,	90–158,	87–158,	4–72,			
		166–234	166–234	163–234	5–72			
	**12052812**	50–107	52–107	50–105	4–72,		30	
					4–72,			
					5–72			
	44662819	4–72	4–70	3–70	4–72,		30	
					4–72,			
					5–72			
	37589137	4–72	4–70	3–70	4–72,		30	
					4–72,			
					5–72			
	57997480	21–87	20–87	17–87	4–72,		36	
					4–72,			
					5–72			
PARK2	**5912028**	14–82,	14–82,	11–82,		4–72,		30
		90–158,	90–58,	87–58,		4–72,		
		166–234	166–234	163–234		5–72		
	**12052812**	50–107	52–107	50–105		4–72,		29
						4–72,		
						5–72		
	10241759	1–67	1–67	1–65		4–72,		30
						4–72,		
						5–72		

**Table 5:** Mutational analysis of the hypothetical proteins with ubiquitin domains.

Sl. no.	Gi ID	Mutational positions	Function as predicted by protein mutant database (PMD)
1	5912028	Nil	No change
2	37589137	I30V	Stability is retained.
		G35K	Melting temperature at pH 3.0 decreases.
3	44662819	I30V	Stability is retained.
		G35K	Melting temperature at pH 3.0 decreases.
4	57997480	G35S	Melting temperature at pH 3.0 decreases.
		K48R	Increase in morphologic response of cells to canavanine, accumulation of high-molecular-weight ubiquitin conjugates and proteome substrates is observed.
5	12052812	R42L	Ubiquitin adenylate affinity for E1 protein decreases.
		G35K	Melting temperature at pH 3.0 decreases.
6	10241759	R42L	Ubiquitin adenylate affinity for E1 protein decreases.
		G35K	Melting temperature at pH 3.0 decreases.

**Table 6:** Conservation of domain fusions in Parkinson's disease targets and human hypothetical sequences.

KEGG sequence	Hypothetical protein Gi IDs	Region of domain fusion with the target			Sequence identity in the fused region
		Cd01492	Cd01491	UBA1	
UBA1	12053109	13–162	13–162	54–162	52/160 = 32%

**Table 7:** Conservation of domain fusions in Parkinson's disease targets and human hypothetical sequences in cluster-5.

KEGG sequence	Hypothetical protein Gi IDs	Region of domain fusion with the target			Sequence identity in the fused region (%)
		Cd00180	Cd00192	PINK1	
PINK1	57997188	14–288	19–273	271–501	17
	5912043	58–340	63–337	271–501	8
	52545876	147–405	152–388	271–501	18
	1905906	53–303	57–299	271–501	19
	12053281	173–419	173–415	271–501	11
	57997093	199–438	193–433	271–501	19
	3510234	28–287	34–284	271–501	13

**Table 8:** Conservation of domain fusions in Parkinson's disease targets and human hypothetical sequences.

KEGG sequence	Hypothetical protein Gi IDs	Region of domain fusion with the target				Sequence identity in the fused region (%)
		Cd01488	Cd01489	Cd01490	UBA1	
UBA1	63994165	198–377	198–397	197–512	470–671	53
	34304594	1–168	1–169	1–303	470–671	48
	7018436	30–192	30–200	30–419	470–671	31
	7018418	71–368	71–331	71–342	470–671	25
